# The acute effects of cannabis, with and without cannabidiol, on attentional bias to cannabis related cues: a randomised, double-blind, placebo-controlled, cross-over study

**DOI:** 10.1007/s00213-024-06543-7

**Published:** 2024-02-28

**Authors:** Daniel Hall, Will Lawn, Shelan Ofori, Katie Trinci, Anya Borissova, Claire Mokrysz, Kat Petrilli, Michael A. P. Bloomfield, Matthew B. Wall, Tom P. Freeman, H. Valerie Curran

**Affiliations:** 1 Clinical Psychopharmacology Unit, Clinical Educational and Health Psychology, University College London, London, UK; 2 Daniel Hall, Springfield University Hospital, 15 Springfield Drive, London, SW17 0YF UK; 3 Department of Psychology, Institute of Psychiatry Psychology and Neuroscience, King’s College, London, UK; 4 Department of Neuroimaging, Institute of Psychiatry Psychology and Neuroscience, King’s College, London, UK; 5 NIHR University College London Hospitals Biomedical Research Centre, University College Hospital, London, UK; 6 Addiction and Mental Health Group (AIM), Department of Psychology, University of Bath, London, UK; 7 Translational Psychiatry Research Group, Research Department of Mental Health Neuroscience, Division of Psychiatry, University College London, London, UK; 8 Invicro London, Hammersmith Hospital, London, UK

**Keywords:** Cannabis, THC, CBD, Adolescence, Attentional bias

## Abstract

**Rationale:**

Attentional bias to drug-related stimuli is hypothesised to contribute towards addiction. However, the acute effects of Δ9-tetrahydrocannabinol (THC) on attentional bias to cannabis cues, the differential response in adults and adolescents, and the moderating effect of cannabidiol (CBD) are unknown.

**Objectives:**

Our study investigated (1) the acute effects of vaporised cannabis on attentional bias to cannabis-related images in adults and adolescents and (2) the moderating influences of age and CBD.

**Methods:**

We conducted a randomised, double-blind, placebo-controlled, cross-over study where three weight-adjusted vaporised cannabis preparations: ‘THC’ (8 mg THC for a 75-kg person), ‘THC + CBD’ (8 mg THC and 24 mg CBD for a 75-kg person) and PLA (matched placebo). Cannabis was administered on 3 separate days to 48 participants, who used cannabis 0.5–3 days/week: 24 adolescents (12 females, aged 16–17) and 24 adults (12 females, aged 26–29). Participants completed a visual probe task with cannabis cues. Our primary outcome was attentional bias to cannabis stimuli, measured using the differential reaction time to a cannabis vs. neutral probe, on 200-ms trials.

**Results:**

In contrast to hypotheses, attention was directed away from cannabis cues on placebo, and there was a main effect of the drug (*F*(2,92) = 3.865, *p* = 0.024, η^2^_*p*_ = 0.077), indicating THC administration eliminated this bias. There was no significant impact of CBD nor an age-by-drug interaction.

**Conclusions:**

Acute THC intoxication eliminated attentional bias away from cannabis cues. There was no evidence of differential response in adolescents compared to adults and no evidence that a moderate vaporised dose of CBD altered the impact of cannabis on attentional bias.

**Trial registration:**

This study was listed with the US National Library of Medicine and registered on ClinicalTrials.gov, URL: Do Adolescents and Adults Differ in Their Acute Response to Cannabis?—Full Text View—ClinicalTrials.gov, registration number: NCT04851392.

## Introduction

Cannabis was the most commonly used drug by adults (7.8%) and 16–24-year-olds (18.7%) in England and Wales in 2019 (The Office of National Statistics 2020, NHS Digital 2019). Public health concerns have arisen as legal cannabis markets flourish along with the emergence of potent cannabis strains associated with adverse health outcomes (Hall and Lynskey 2020; Wilson et al. 2019; Petrilli et al. 2022). Adolescent cannabis use has been associated with a wide range of adverse cognitive, educational, and mental health outcomes (Hall et al. 2020; Wilson et al. 2019).

THC (Δ9-tetrahydrocannabinol) is a CB1 receptor partial agonist responsible for cannabis’ intoxicating effects, whereas CBD (cannabidiol) has multiple mechanisms of action, including as a negative allosteric modulator of the CB1 receptor (Freeman et al. 2019). Adolescence may be a critical developmental period that cannabis use can perturb; concentrations of the endocannabinoid 2-arachidonoylglycerol and cannabinoid receptor 1 (CB1) are known to peak throughout mesocorticolimbic structures before declining in adulthood (Salmanzadeh et al. 2020; Rubino and Parolaro 2016; Meyer et al. 2018; Galve-Roperh et al. 2009). There is significant interest in CBD as a harm-reduction agent congruent with its mechanism of action, but evidence is mixed, with recent studies reporting no consistent evidence of CBD attenuating the effects of THC (Freeman et al. 2019; Englund et al. 2022; Lawn et al. 2023). There is also some emerging evidence in humans that CBD may alter the pharmacokinetics of THC and increase plasma THC levels (Zamarripa et al. 2023; Nadulski et al. 2005; Lawn et al. 2023).

Similarly, there is mixed evidence of differential pharmacodynamic effects in adults and adolescents during acute cannabis intoxication. When acutely intoxicated with cannabis, Mokrysz et al. (2016) reported that adolescents had reduced psychotic-like experiences, diminished anxiety and improved item recall compared to adults. Murray et al. (2022) reported a greater impact of acute cannabis on a response inhibition task in adolescence along with EEG changes. However, Lawn et al. (2023) and Skumlien et al. (2022), partner analyses from the same dataset as this current article, found no differences in reward processing, psychotomimetic nor memory-impairing and subjective effects. The evidence so far suggests that there are some effects on cognition of acute cannabis intoxication that may differ between adolescents and adults, but these are not always replicated. However, it is not known if cognitive processes related to attention might be differentially affected, by cannabinoids, age and the nature of the attended stimulus.

Attentional bias is a cognitive process whereby selective attention is focussed automatically towards appetitive or aversive environmental stimuli, thereby initiating behavioural changes such as increased arousal, orientation, substance-craving and approach behaviour (Field and Cox 2008; Wiers and Stacy 2006). These attentional and behavioural changes are typically described as ‘implicit’, meaning they are involuntary, automatic and not measurable by self-report (Wiers et al. 2007). Substance users consistently display attentional bias to substance cues (Field and Cox 2008; Zhang et al. 2018). The visual probe task is a commonly used experimental measure of attentional bias that involves the presentation of a pair of visual stimuli and the measurement of a manual response time (Field and Cox 2008). Short stimulus exposure (< 200 ms) permits sufficient time to shift attention once within a single ocular saccade and emphasises the initiation of attention (Field and Cox 2008; Rooijen et al. 2017). Long stimulus exposures (500–2000 ms) place greater emphasis on maintenance and disengagement of attention as long times allow for multiple saccades and shifting of attention repeatedly. There is on-going debate about the reliability of attentional bias as a psychometric measure in addiction (Christiansen et al. 2015; Jones et al. 2018). The reliability of visual probe trials can be improved by the use of short stimulus times, increased number of cues, repeated measures in participants and re-scaling of outliers (Jones et al. 2018; Price et al. 2015).

A recent meta-analysis of cannabis attentional bias tasks reported that users paid closer attention to cannabis cues compared to controls, especially visual cues (O'Neill et al. 2020). Attentional bias tends to be more apparent in heavier users with severe cannabis use disorder (CUD) but is also detectable in non-dependent users (Kroon et al. 2022; Cousijn et al. 2013). Attentional bias has been hypothesised to be associated with craving and increased problem use of cannabis in less severe users (Kroon et al. 2022). Also, shorter exposure times tend to elicit attentional bias in cannabis users (Zhang et al. 2020; O'Neill et al. 2020). Adolescents are hypothesised to be more vulnerable to substance cues due to the lack of inhibitory regulation by an immature prefrontal cortex (Wiers et al. 2007; Cousijn et al. 2015). There are no studies comparing attentional bias directly in adult and adolescent substance users, but evidence in adolescents alone suggests, that like adults, they have increased attentional bias for substances (Wiers et al. 2015; Hemel-Ruiter et al. 2016; Cousijn et al. 2015).

There is only one study looking directly at the acute effects of cannabis on attentional bias using a visual probe paradigm. Morgan et al. (2010) performed a naturalistic study with adolescents and young adults aged 16–24 who were allowed to smoke a typical amount of their own, usual cannabis and then perform a visual probe task. Participants were grouped by high CBD content (> 1%) and low CBD content (< 1%) with results indicating increased attentional bias to food and drug-related stimuli at short exposure times (250 ms) for low CBD participants but no attentional bias in high CBD participants. Some research looking at the acute effects of CBD have found that moderate to high doses of CBD (e.g. 800 mg oral CBD) may reduce attentional bias to drug cues (Hindocha et al. 2018; Prud'homme et al. 2015). However, to our knowledge, no previous studies have investigated the effects of THC and/or CBD on attentional bias in a randomised, placebo-controlled design.

We do not know if acute THC enhances attentional bias towards cannabis-related cues. Furthermore, although unknown, there is reason to hypothesise that adolescents may be more vulnerable to the appetitive effects of THC and that co-administered CBD may be protective. This study therefore aimed to examine the acute effects of cannabis with and without CBD on attentional bias to cannabis stimuli in adolescents and adults. We hypothesised across age group that (1) there would be a main effect of the drug such that active cannabis (both THC and THC + CBD) would increase attentional bias to cannabis-related stimuli as compared with placebo and (2) the addition of CBD to THC would attenuate participants’ attentional bias to cannabis-related images, as compared with THC alone, and (3) we conducted exploratory analyses whether there is an age-by-drug interaction, such that active cannabis vs. placebo might increase attentional bias differently in adolescents and adults.

## Method

### Study design

CannTeen Acute was a randomised, single-centre, double-blind, placebo-controlled, cross-over study performed in London, UK (Lawn et al. 2021). Here, we report secondary outcomes for this study (primary outcomes are reported in Lawn et al. (2023)). Participants attended three experimental sessions and received one of three drug conditions, either placebo (PLA), THC alone or THC + CBD allocated via block randomisation. Experimental sessions were separated by a minimum of 1 week to allow washout. The study was registered on ClinicalTrials.gov (NCT04851392), and the protocol was pre-registered with Open Science Framework (Hall and Lawn 2022)**.** The study was approved by the UCL Ethics Committee (project code: 5929/005), and all participants provided informed, written consent. All research was conducted in accordance with the Declaration of Helsinki.

### Participants

The study recruited on a ‘per-protocol’ basis, such that recruitment continued until a total of 48 participants had completed all three sessions, and non-completers were excluded from the final analysis. There were 24 adults (12 female, ages 26–29 years) and 24 adolescents (12 female, ages 16–17 years) matched on the frequency of cannabis use (0.5–3 days/week). Participants were recruited via online advertisement and word of mouth. Adult cannabis users were excluded if they had used cannabis regularly prior to age 18. Exclusion criteria included any current or historical medical/psychiatric illness that the study doctor considered exclusionary, being a current recipient of mental health treatment, any current administration of psychotropic drugs likely to interfere with dependent variables or cannabis administration, greater than twice per month use of illegal drugs other than cannabis, being pregnant or breastfeeding, a personal or family history of psychosis, a DSM-5 diagnosis of severe cannabis use disorder, nicotine dependence (defined by a heaviness of smoking score > 1), and contraindication to magnetic resonance imaging. Participants were screened using an online questionnaire and telephone follow-up before being invited to take part in-person (pre-COVID) or virtual (post-COVID) baseline session where eligibility was fully assessed. Our participants were regular, non-heavy and non-dependent cannabis users—a common pattern of use.

### Power analysis

This experiment was powered to detect an age-group-by-drug interaction on the primary outcome, psychotic-like symptoms, reported elsewhere (Lawn et al. 2021). However, with an alpha threshold of 0.05, we have 80% power to detect a within-subjects main effect (drug) and a within-between interaction (age group by drug) with an effect size of Cohen’s *f* = 0.24 or greater.

### Drug administration

Cannabis was acquired from the Dutch company Bedrocan (https://bedrocan.com/) and imported under a UK Home Office licence. In the experiment, three dried-flower cannabis products were used to make our cannabis preparations, ‘Bedrocan’ cannabis (20.2% THC and 0.1% CBD), ‘Bedrolite’ cannabis (0.4% THC and 8.5% CBD) and ‘Bedrobinol’ placebo cannabis (0% THC and 0% CBD). Placebo cannabis was designed to resemble active cannabis due to its terpene content, taste and aroma. Placebo cannabis terpene profile was quality assured as equivalent to active cannabis, and the profile is available on the manufacturer’s website. Doses for participants were calculated based on their weights which were checked at the start of every session to ensure that doses remained valid. Cannabis preparations were administered such that each participant would receive 0.107 mg/kg of THC for the ‘THC’ condition (equivalent to 8 mg of THC, or 1.6 standard units of THC, for a 75-kg adult) and 0.107 mg/kg of THC and 0.320 mg/kg of CBD for the ‘THC + CBD’ condition (equivalent to 8 mg of THC and 24 mg of CBD for a 75-kg adult). This dose of THC equates to approximately one-quarter of a typically consumed ‘joint’ in the UK (Freeman et al. 2014). The weight of each of the three cannabis preparations was held constant using placebo cannabis adjustments. Drugs were blinded to participants and experimenters. Cannabis was vaporised at 210 °C using a volcano medic vaporiser (Storz and Bickel, Tuttlingen, Germany) and inhaled by participants in two consecutive 9-min sessions using a standardised procedure (Lawn et al. 2023).

### Visual probe task

**Fig. 1:**
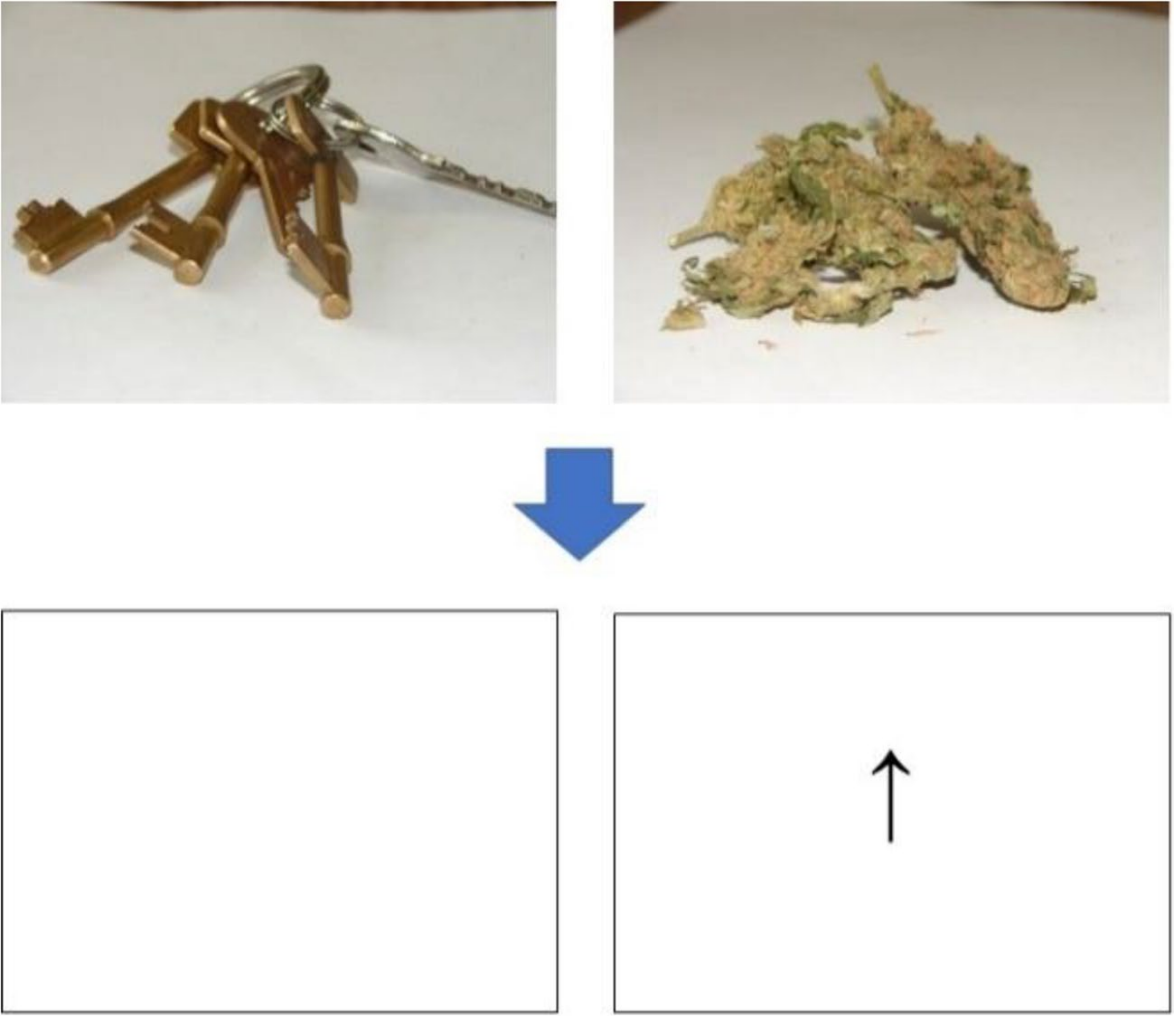
Visual representation of dot-probe task with a pair of matched simultaneous images (one neutral and the other either cannabis or food-related) presented for either 200 ms or 500 ms, with a single image then replaced by an up or down arrow

A visual probe task was used to assess attentional bias, adapted from a previous study (Morgan et al. 2010). Each trial began with a 500-ms visual fixation point. The participant was then presented simultaneously with a pair of images side by side that would appear for a presentation time of either a short 200-ms or a long 500-ms duration. The image pair contained either a cannabis or food-related image along with a matched neutral image. Images were matched in terms of composition and brightness (see Fig. 1 for an example image pair). Drug stimuli were images of cannabis and its use familiar to cannabis users, e.g. images of cannabis, cannabis products and joints being prepared and lit. The use of ecologically valid substance cues can help improve internal consistency in visual probe tasks (Jones et al. 2018). After the presentation time, one of the image pairs was replaced by a visual probe, either an up or down arrow. The participant had to respond to the probe’s orientation by pressing an appropriate up or down arrow response on the keyboard as quickly and accurately as possible. The probe would remain on screen until a correct response was made by the participant. Probes replaced stimulus and neutral images equally.

**Fig. 2:**
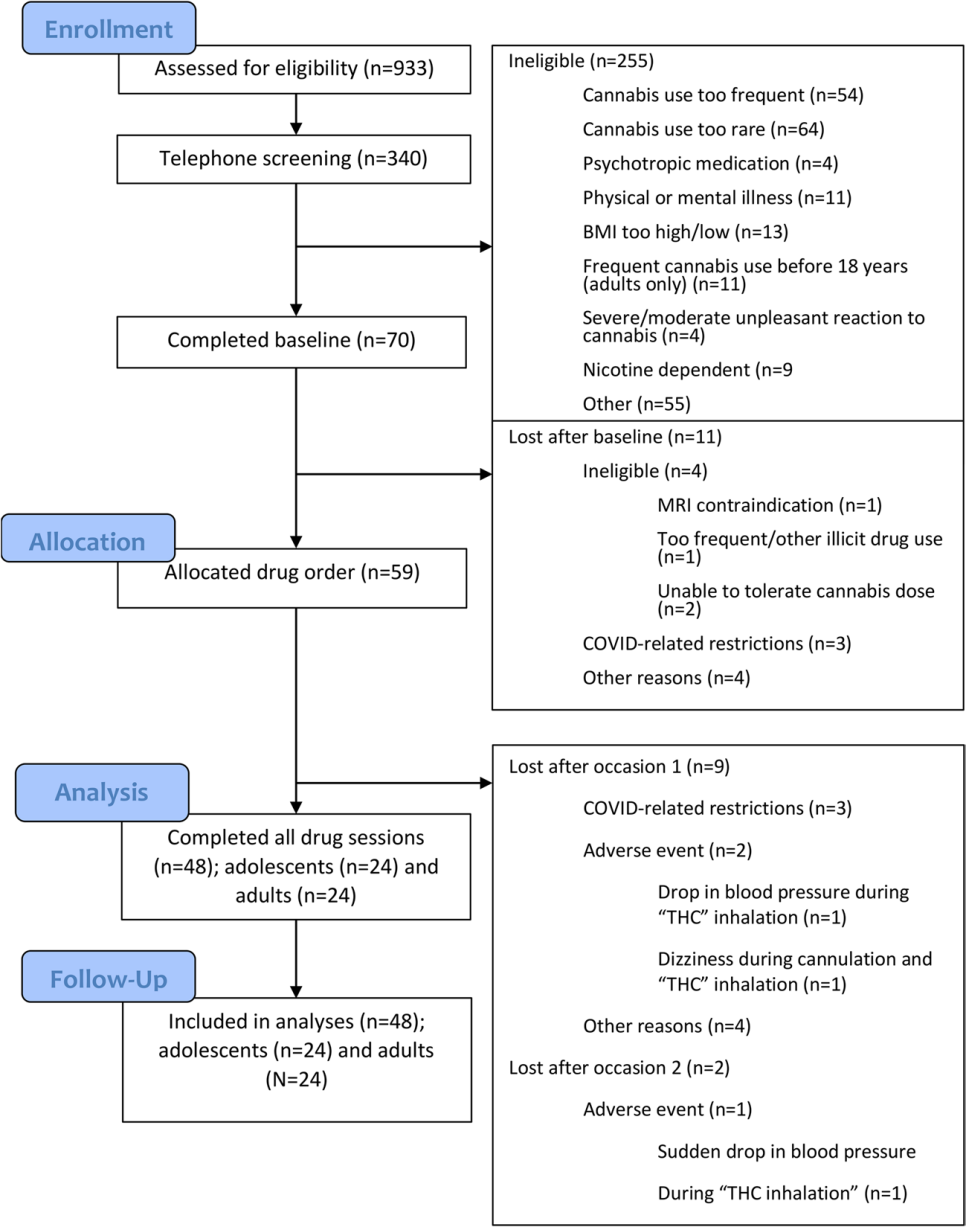
Consolidated standards or reporting trials (CONSORT) flowchart for all participants

There was a total of 112 experimental trials. Trial order was randomised each time the task was run. There were fourteen image pairs in total split equally by cannabis and food, each stimulus image with its own companion neutral image. Each pair of images was repeated 8 times. The appearance of each individual image on the left and right of the screen, the appearance of the probe behind neutral images and stimulus-related images and the presentation time of each image pair were equally balanced. Visual bias scores were calculated by subtracting the mean reaction time for the drug stimulus from the mean reaction time to the neutral stimulus. A more positive score indicated a faster reaction time and greater attentional bias to either the drug or food stimulus, e.g. cannabis attentional bias_200ms_ = RT_average_neutral_200ms_ − RT_average___cannabis_200ms_. A more negative score indicated attentional bias away from the stimulus, and a positive score indicated attentional bias towards the image. Although the task included food data, this was not analysed Fig. 2.

### Procedure

Participants attended three separate drug administration sessions for each drug condition (placebo, THC and THC + CBD), with sessions typically lasting 5–6 h (for total experimental procedure see Lawn et al. (2023)). Sessions were separated by 1 week to allow adequate drug washout. All participants were instructed to refrain from all illicit drug use (including cannabis) 72 h before each session and alcohol use 24 h before each session. Participant sobriety and eligibility were tested at the start of each session by self-report of substance use, alcohol testing via breathalyser and a salivary drug screen. The visual probe task was performed an average of 2 h 13 min (min. 1 h 53 m–max. 2 h 55 m) after initiation of drug inhalation.

### Statistical analysis

All statistical analysis was carried out using IBM SPSS Statistics Software Version 27.0. The primary outcome variable was the cannabis visual bias scores for 200 ms stimuli based on previous research suggesting that short presentation times elicit attentional bias in cannabis users along with increased reliability of the visual probe task (Zhang et al. 2020; O'Neill et al. 2020; Jones et al. 2018; Hindocha et al. 2018). Outliers were re-scaled using winsorization to improve reliability and were replaced by the next highest value within 1.5 times the inter-quartile range (Price et al. 2015). All participants were included in the analysis. Data were visually inspected for normality, and parametric tests were subsequently used.

One sample *t*-tests were used to investigate the direction of attentional bias in each drug condition prior to ANOVA analysis. For our primary analysis, a 3 × 2 ANOVA was performed with within-subject factors of drug type (PLA vs. THC vs. THC + CBD) and between-subject factors of age group (adolescents vs. adults) for cannabis-related visual bias scores during 200-ms trials. If sphericity was violated, then Greenhouse-Geissner corrected *F*-values and *p*-values were reported. Any main effects or interaction effects discovered on ANOVA testing were explored using post hoc Bonferroni corrected *t*-tests. Finally, we conducted a 3 × 2 ANOVA for the long 500-ms trials of cannabis image pairs, with a within-subject factor of the drug (PLA vs. THC vs. THC + CBD) and between-subject factor of age group (adolescents vs. adults).

## Results

### Participant demographics

Table 1 provides a full summary of participant characteristics. Participants were overall well matched on gender and current cannabis use with no significant differences in cannabis use frequency (adolescents 1.41 days/week, SD = 0.77 and adults 1.45 days/week, SD = 0.77) and no differences in the time since cannabis last used or amount of cannabis used. As expected from the design of the study, adults had started using cannabis later and had used it for more lifetime days than adolescents.

**Table 1:** Summary of participant baseline socio-demographic characteristics and baseline drug use

	Adolescent (*n* = 24)	Adult (*n* = 24)
Gender		
Female	12 (50.0%)	12 (50.0%)
Male	12 (50.0%)	12 (50.0%)
Age (years)	17.17 (0.43)	27.77 (1.04)*
Ethnicity		
White	17 (70.8%)	18 (75.0%)
Mixed	4 (16.7%)	1 (4.2%)
Asian	1 (4.2%)	3 (12.5%)
Black	0 (0%)	2 (8.3%)
Other	1 (4.2%)	0 (0%)
Prefer not to say	1 (4.2%)	0 (0%)
Adjusted WTAR Score	110.96 (12.46)	117.83 (6.12)*
SES		
Mother’s education below undergraduate degree	8 (33.3%)	8 (33.3%)
Mother’s education undergraduate degree or above	16 (66.7%)	16 (66.7%)
BDI	10.38 (8.55)	5.29 (6.45)*
BAI	10.42 (8.15)	6 (4.92)*
CTQ-25	35.74 (9.29) (*n* = 23)	37 (10.7)
Short-UPPS-P	48.17 (7.51)	42.75 (8.87)*
Alcohol use frequency (days/week)	0.56 (0.62)	2.16 (1.7)***
AUDIT	5.88 (5.39)	7.71 (4.59)
Cigarette use frequency (days/week)	1.6 (2.25)	0.6 (1.3)
Other illicit drug use frequency (days/month)	0.46 (0.83)	0.33 (0.87)
Other illicit drug^No^	22 (91.7%)	22 (91.7%)
Use: monthly use^Yes^	2 (8.3%)	2 (8.3%)
Cannabis: lifetime days of use	153.67 (89.97)	544.29 (630.94)**
Cannabis: age of first use (years)	14.55 (1.03)	18.17 (2.62)***
Cannabis: use frequency (days/week)	1.41 (0.77)	1.45 (0.77)
Cannabis: time since last use (days)	7.8 (7.34)	5.06 (2.79)
Cannabis: amount used (grammes)	0.81 (0.56)	0.5 (0.52) (*n* = 23)
CUDIT-R	10.17 (3.14)	7.21 (3.31)**

For continuous data, mean (SD) is shown and for categorical data (%).Total DSM CUD number of symptoms 1.42 (1.69) 0.92 (1.35)

*SES* socioeconomic status, *AUDIT* alcohol use disorders identification test, *BDI* Becks depression inventory, *CUDIT-R* cannabis use disorder identification test-revised, *s-UPPS-P* short urgency-premeditation-perseverance-sensation seeking-positive urgency, *WTAR* Weschler test of adult reading

^*^p < 0.05, **p < 0.01, ***p < 0.001

### Primary outcome—attentional bias to cannabis-related images at 200 ms

**Fig. 3:**
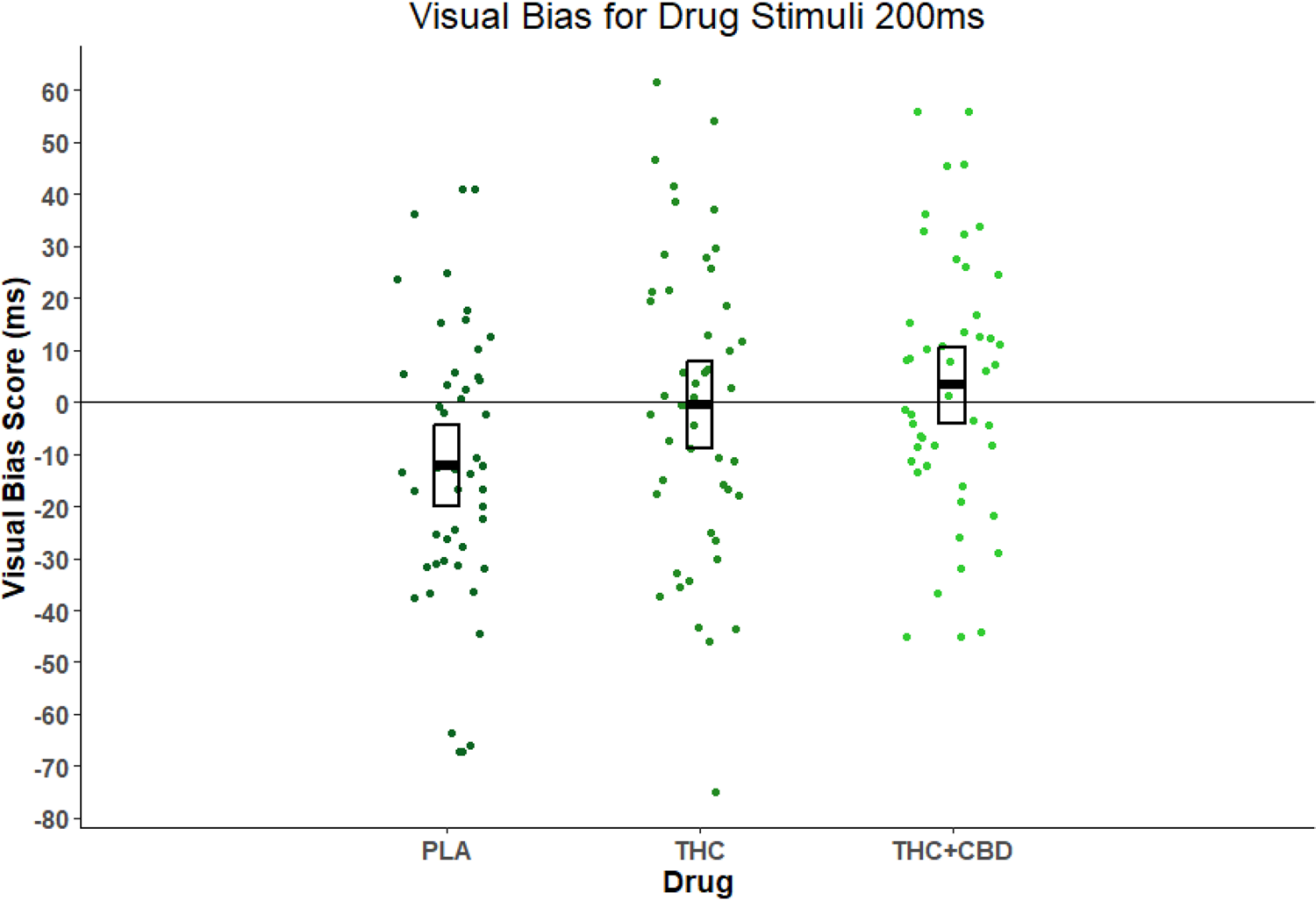
Individual mean visual bias scores for each drug condition (*n* = 48) and all ages, with crossbars indicating mean and 95% confidence intervals (where PLA = placebo, THC = tetrahydrocannabinol, and THC + CBD = tetrahydrocannabinol and cannabidiol). The main effect of drug was significant *p* = 0.022. Post hoc tests corrected for multiple comparisons showed THC + CBD > PLA *p* = 0.040

Table 2 provides descriptive statistics for attentional bias to cannabis stimuli at the 200-ms presentation time. There was a significant main effect of drug (*F*(2,92) = 3.991, *p* = 0.022, η^2^_*p*_ = 0.080), but there was no main effect of age (*F*(1,46) = 0.272, *p* = 0.604, η^2^_*p*_ = 0.006) and no age-by-drug interaction (*F*(2,92) = 0.715, *p* = 0.402, η^2^_*p*_ = 0.015). The significant main effect of drug appears to have been driven by THC + CBD having greater attentional bias than PLA (*t*(47) = 2.577, *p* = 0.040, MD = 15.436). There was no significant effect of THC vs. PLA (*t*(47) = 2.134, *p* = 0.115, MD = 11.724) nor THC vs. THC + CBD (*t*(47) = 0.661, *p* = 1.000, MD =  − 3.712). Figure 3 shows the main effect of the drug for all participants. For the 200-ms presentation, there was a negative attentional bias on ‘PLA’ (score =  − 12.27, *p* < 0.05), i.e. participants were faster when the probe was behind the control image than the cannabis picture (participants looked away from cannabis pictures). However, following ‘THC + CBD’ (score = 3.63, *p* > 0.05) and ‘THC’ (score =  − 0.54, *p* > 0.05), the attentional bias was eliminated.

**Table 2:** Descriptive statistics for visual bias scores at 200 ms presentation time. For one sample *t*-tests performed on means †*p* = 0.059, *p < 0.05, **p < 0.01

	Adults	Adolescents	Both combined
	Mean (SD) (median, min–max)	Mean (SD) (median, min–max)	Mean (SD) (median, min–max)
PLA	− 12.27* (23.39) (− 12.78, − 65.90–40.88)	− 12.26† (30.26) (− 15.13, − 67.29–40.88)	− 12.27** (26.76) (− 13.22, − 67.29–40.88)
THC	1.21 (28.42) (1.20, − 74.98–46.57)	− 2.30 (29.85) (0.12, − 45.83–61.58)	− 0.54 (28.88) (0.22, − 74.98–61.58)
THC + CBD	− 1.90 (27.99) (− 3.02, − 44.97–55.94)	8.23 (21.60) (7.81, − 26.10–55.94)	3.17 (25.25) (3.63, − 44.97–55.94)

### Secondary outcome—attentional bias to cannabis-related images at 500 ms

Table 3 provides descriptive statistics for attentional bias to cannabis-related stimuli at 500-ms presentation time. For attentional bias to drug-related stimuli at 500-ms presentation time, there was no significant effect of drug (*F*(2,92) = 1.148, p = 0.322, η^2^_*p*_ = 0.024), nor age (*F*(1,46) = 2.641, *p* = 0.111, η^2^_*p*_ = 0.054) and nor age-by-drug interaction (*F*(2,92) = 0.198, *p* = 0.658, η^2^_*p*_ = 0.004).

**Table 3:** Descriptive statistics for attentional bias at 500 ms presentation time. For one sample *t*-tests performed on means *p < 0.05, **p < 0.01

	Adults	Adolescents	Both combined
	Mean (SD) (median, min–max)	Mean (SD) (median, min–max)	Mean (SD) (median, min–max)
PLA	− 3.32 (36.17) (− 1.53, − 81.80–62.17)	− 13.64* (30.20) (− 6.55, − 81.08–62.17)	− 8.48 (33.37) (− 4.41, − 81.08–62.17)
THC	− 17.47* (41.45) (− 18.39, − 96.63–57.36)	− 16.90* (34.70) (− 17.04, − 96.63–41.44)	− 17.19*(37.81) (− 17.14, − 96.63–57.36)
THC + CBD	0.32 (20.44) (1.14, − 32.89–39.54)	− 15.77* (32.98) (− 21.62, − 63.83–40.44)	− 7.72 (28.33) (− 9.95, − 63.83–40.44)

## Discussion

This study was the first randomised, double-blind, placebo-controlled trial to investigate the acute effects of THC and THC + CBD using a visual probe task in adults and adolescents. For our primary outcome at 200-ms presentation times, we found a main effect of the drug across all age groups indicating that visual bias away from cannabis-related stimuli under placebo conditions was eliminated by acute intoxication with active cannabis (THC or THC + CBD). We found that this effect was primarily driven by THC + CBD. No changes in attentional bias were detected at 500-ms exposure times.

This result is similar to Morgan et al. (2010) whose low-CBD-to-THC group elicited a positive attentional bias to drug-related stimuli at 250-ms presentation times; however, they did not have a placebo control. Our participants had an initial attentional bias away from cannabis cues under placebo conditions that was eliminated by acute THC intoxication. Only people with severe cannabis use disorder, a population we excluded, tend to have baseline positive attentional bias to cannabis cues (Kroon et al. 2022). Attentional bias, however, is still of relevance in mild to moderate CUD as a potential indicator of people vulnerable to developing severe CUD and dependence. Our results suggest that acute THC intoxication increased the appetitive qualities of cannabis cues. This effect does not appear particularly strong in 1.5/day week cannabis users without severe CUD but was present and could be relevant in driving further cannabis use in acutely intoxicated users.

The lack of age-by-drug interactions suggests that adolescents (aged 16–17) do not differ from adults (aged 26–29) in attentional bias to cannabis-related cues when acutely intoxicated. Our participants tended to be older adolescents, and it has been proposed that attentional bias to reward cues in adolescence is only present in the initiation stage of substance misuse and at younger ages (Hemel-Ruiter et al. 2016). Other studies of acute cannabis intoxication comparing adults and adolescents have reported no detectable differences on a range of measures (Lawn et al. 2023; Skumlien et al. 2022). Similar findings have been reported between adults and adolescents (16–17 years) with alcohol use disorder, where no difference in attentional bias, craving and approach behaviour was elicited (Cousijn et al. 2020).

Again, contrary to our hypothesis, CBD had no moderating effect on attentional bias. Morgan et al. (2010) in their naturalistic study reported that a high CBD-to-THC ratio in cannabis diminished attentional bias to drug-related cues at 250 ms. However, their participants were slightly older and heavier cannabis users. Also, their naturalistic study had very different conditions to this one; for example, they were tested after using their own cannabis at a dose they liked and had donated a sample for THC and CBD concentration analysis. Evidence regarding CBD’s moderating effect is mixed (Freeman et al. 2019; Prud'homme et al. 2015). A recent randomised-controlled study using vaporised cannabis found no modulating effect of up to 30 mg of CBD on THC’s cognitive or psychotic effects (Englund et al. 2022). It may be that significantly larger doses of CBD or IV administration are necessary (Hindocha et al. 2018; Englund et al. 2013). For example, in a phase 2a clinical study for people with severe CUD, daily doses of 400 mg and 800 mg of CBD were helpful in assisting reduction in use but 200 mg was no better than placebo (Freeman et al. 2020).

This study had significant strengths, primarily that it was a double-blind, randomised and placebo-controlled trial with biologically confirmed abstinence before each experimental session. This study is one of the largest attentional bias studies with acute substance intoxication performed; however, it was not powered specifically for a visual probe task. Although concerns about the reliability of visual probe tasks have generated on-going debate, they remain widely used due to their validity and pragmatic use (Christiansen et al. 2015; Jones et al. 2018). We did not use any other correlates of attentional bias such as eye-tracking, but several aspects of the study design increase reliability, the use of repeated measures, the high number of trials and the statistical methods chosen.

In this randomised, placebo-controlled cross-over study, acute THC administration eliminated attentional bias away from cannabis-related cues presented at 200 ms across all age groups. This has potential implications for cannabis consumption and addiction. There were no significant age-by-drug interactions and no significant moderating effect of CBD. This study adds to the growing body of evidence that adolescents are neither more vulnerable nor more resilient to the acute intoxicating effects of cannabis. Also, consistent with current evidence, this study indicated that vaporised CBD had no significant moderating effect on attentional bias due to acute THC intoxication at this dose.
